# Probing the cw-Laser-Induced Fluorescence Enhancement in CsPbBr_3_ Nanocrystal Thin Films: An Interplay between Photo and Thermal Activation

**DOI:** 10.1021/acsami.4c03934

**Published:** 2024-06-17

**Authors:** Gabriel Fabrício de Souza, Letícia Ferreira Magalhães, Thaís Adriany de Souza Carvalho, Diego Lourençoni Ferreira, Richard Silveira Pereira, Thiago Rodrigues da Cunha, Jefferson Bettini, Marco Antônio Schiavon, Marcelo Gonçalves Vivas

**Affiliations:** † Laboratório de Espectroscopia Óptica e Fotônica, Universidade Federal de Alfenas, 37715-400 Poços de Caldas, MG, Brazil; ‡ Grupo de Pesquisa em Química de Materiais, Universidade Federal de São João del-Rei, 36301-160 São João del-Rei, MG, Brazil; § Laboratório Nacional de Nanotecnologia, Centro Nacional de Pesquisa em Energia e Materiais, 13083-970 Campinas, São Paulo, Brazil

**Keywords:** perovskite nanocrystals, thin films, hyperspectral fluorescence microscopy, finite-difference method, fluorescence enhancement

## Abstract

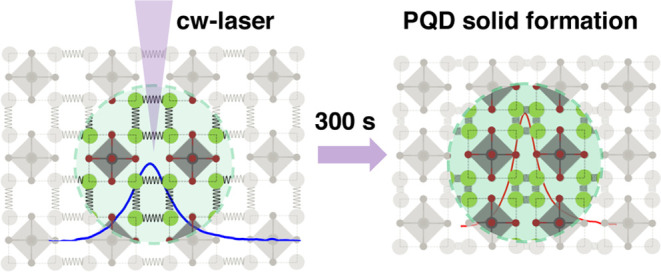

Perovskite nanocrystals hold significant promise for a wide range of applications, including solar cells, LEDs, photocatalysts, humidity and temperature sensors, memory devices, and low-cost photodetectors. Such technological potential stems from their exceptional quantum efficiency and charge carrier conduction capability. Nevertheless, the underlying mechanisms of photoexcitation, such as phase segregation, annealing, and ionic diffusion, remain insufficiently understood. In this context, we harnessed hyperspectral fluorescence microspectroscopy to advance our comprehension of fluorescence enhancement triggered by UV continuous-wave (cw) laser irradiation of CsPbBr_3_ colloidal nanocrystal thin films. Initially, we explored the kinetics of fluorescence enhancement and observed that its efficiency (φ_ph_) correlates with the laser power (*P*), following the relationship φ_ph_ = 7.7⟨*P*⟩^0.47±0.02^. Subsequently, we estimated the local temperature induced by the laser, utilizing the finite-difference method framework, and calculated the activation energy (*E*_a_) required for fluorescence enhancement to occur. Our findings revealed a very low activation energy, *E*_a_ ∼ 9 kJ/mol. Moreover, we mapped the fluorescence photoenhancement by spatial scanning and real-time static mode to determine its microscale length. Below a laser power of 60 μW, the photothermal diffusion length exhibited nearly constant values of approximately (22 ± 5) μm, while a significant increase was observed at higher laser power levels. These results were ascribed to the formation of nanocrystal superclusters within the film, which involves the interparticle spacing reduction, creating the so-called quantum dot solid configuration along with laser-induced annealing for higher laser powers.

## 1 Introduction

Great advances have been achieved in the past decade concerning perovskite nanocrystals, an emerging class of semiconductors comprising different compositions under the general formula ABX_3_ (A and B are cations of different sizes and X is an anion).^1−3^ In particular, fully inorganic perovskite nanocrystals based on the cesium lead halides CsPbX_3_ (X = Cl^–^, Br^–^, I^–^) arouse interest as promising optoelectronic materials because of their excellent luminescence properties, high photoluminescence quantum yields (50–90%), high absorption coefficient, and remarkable charge carrier mobility.^4−7^ In addition, the bandgap of these nanosized semiconductors can be tuned across the visible spectrum to near-infrared by controlling the halide composition (X).^1,2,8^ Due to these properties, mixed lead halide perovskites (MLHPs) reveal many possibilities in photovoltaic applications, with a rapid increase in energy conversion efficiency being observed in perovskite solar cells in recent years (4–26%; National Renewable Energy Laboratory),^9^ making them stand out in the emerging generation of photovoltaics.^5,10−12^

Despite these qualities, achieving long-term operational stability and increasing the conversion efficiency of MLHP-based photovoltaic devices are still challenging due to the soft lattice and carrier recombination.^13^ Recently, many studies have reported phenomena related to UV laser-induced phase segregation and ionic diffusion in 3D and 2D perovskites.^14−20^ These processes affect the photovoltaic performance because they cause the accumulation of ions on the electrode surface, leading to the effects of polarization, hysteresis, and degradation of halides.^15^

On the other hand, the continuous-wave (cw) laser can also improve the optical features of perovskite crystals and nanocrystal films. In this context, You et al.^11^ explored the effects of a cw-laser with excitation wavelengths of 405, 450, and 660 nm on ultrafast laser annealing of mixed perovskite films comprising methylammonium lead iodide (MAPbI_3_) and (CsPbI_3_)_0.05_(FAPbI_3_)_0.95_(MAPbBr_3_)_0.05_. Their findings revealed a notable enhancement in the efficiency of perovskite solar cells following laser-induced annealing. This improvement was attributed to the increased crystallinity and light absorption capacity of the perovskite films and the selective growth of larger perovskite grains facilitated by the rapid annealing process induced by the laser. More recently, Ji et al.^21^ demonstrated that continuous-wave laser irradiation at 405 nm boosted the fluorescence amplitude of CsPbBr_3_ nanocrystal thin films. This enhancement was ascribed to improved thin film uniformity and density. Atomic force microscopy (AFM) analysis indicated that the laser treatment led to a reorganization of grain structures and reduced defects within the film. Another recent study^22^ investigated the photostability of CsPbBr_3_ colloidal nanocrystals in solution under continuous UV illumination. It was hypothesized that the UV photons act on the nanocrystal surface, modifying their stability, which depends on the equilibrium between desorption and adsorption of the surface ligands. Thus, utilizing laser treatment on thin films of CsPbBr_3_ nanocrystals could create improved light emission diodes, high-resolution displays, lasers, and photovoltaic devices.

Nevertheless, none of the mentioned studies investigated the kinetic and thermodynamic processes underlying the photoinduced fluorescence enhancement in nanocrystalline perovskite thin films. Such a phenomenon will be addressed in the present paper by using a suitable combination of experimental and theoretical methods. Thus, herein, we aim to explore and correlate a series of physical parameters associated with the fluorescence enhancement in spin-coated CsPbBr_3_ nanocrystal thin films subject to UV laser irradiation. For this purpose, the hyperspectral fluorescence microscopy technique is initially used to monitor the laser-induced fluorescence kinetics over the excitation time and space (fluorescence image). Subsequently, the generated kinetic data (photoenhancement rates at distinct laser powers) are then correlated with thermodynamic properties (temperature locally induced by the incident laser and photothermal diffusion length) through a computational approach based on the solution of the Fourier heat equation via the finite difference method and on Fick’s second law of diffusion.

## 2 Experimental Section

### 2.1 Colloidal Synthesis of CsPbBr_3_ Perovskite Nanocrystals

#### 2.1.1 Materials

Cesium carbonate (Cs_2_CO_3_, Sigma-Aldrich, 99,9%), lead(II) bromide (PbBr_2_, Sigma-Aldrich, 99%), oleic acid (OA, Sigma-Aldrich, 90%), 1-octadecene (ODE, Sigma-Aldrich, 90%), oleylamine (OAm, Sigma-Aldrich, 90%), isopropyl alcohol (Dinâmica, 99,5%), and toluene (Synth, 99,5%) were used.

#### 2.1.2 Preparation Methods

The chemical synthesis procedures performed in the present work were adapted from those reported by Protesescu et al.^2^ Briefly, cesium oleate (Cs-oleate) was prepared by adding 0.0814 g of Cs_2_CO_3_, 4 mL of ODE, and 0.25 mL of OA in a three-neck flask and the resulting mixture was dried under vacuum for 1 h at 120 °C. Then, argon was injected into the flask at 150 °C until the complete reaction of Cs_2_CO_3_ with OA. To prepare the CsPbBr_3_ NCs, 0.069 g of PbBr_2,_ 5.0 mL of ODE, 0.5 mL of OA, and 0.5 mL of OAm were added in a three-neck flask, and the mixture was dried under vacuum at 120 °C for 1 h. Past this period, the solution was heated to 150 °C under an Ar atmosphere. Soon after, 0.4 mL of Cs-oleate was quickly injected and, after 10 s, the solution was cooled in an ice bath for 5 s. To purify the NCs, the suspension was transferred to a Falcon tube and isopropyl alcohol was added. The tube was then taken to the centrifuge for 30 min at 9000 rpm. Finally, the supernatant was discarded, and the particles were dispersed in toluene.

### 2.2 Fabrication of Perovskite Films

Perovskite thin films were prepared by spin coating. For this purpose, microscope blades were used as substrates for material deposition. These blades were cleaned in ultrasonic baths of ultrapure water, ethanol, isopropanol, and acetone, each wash lasting for 10 min. Then, they were taken to the Plasma Cleaner for 10 min to remove organic contaminants. After drying at room temperature, the previously prepared CsPbBr_3_ nanocrystal suspension was deposited by rotation on the substrate in two steps: at 400 rpm for 30 s and then at 6000 rpm for 10 s in a spin coater. The thin film was stored in a glovebox under an inert atmosphere for further characterization.

### 2.3 UV–vis Absorption Spectroscopy/Diffuse Reflectance

The thin film analyses were performed on an Agilent Cary 5000 UV–vis–NIR spectrophotometer with a diffuse reflectance accessory (DRA).

### 2.4 Evaluation of the Photoenhancement Effect of CsPbBr_3_ Thin Films Using a UV Lamp

The CsPbBr_3_ films were exposed to continuous light radiation using a 365 nm UV excitation lamp (40 W). Thus, we set the UV-lamp exposure time to observe the fluorescence enhancement before the photobleaching takes place in our experiments. Such time was 10 s. The absorption and fluorescence lifetime measurements were carried out in the same film region where fluorescence enhancement was observed. Time-resolved photoluminescence measurements were performed using a Horiba Fluorolog-3 JobinYvon spectrofluorimeter with a thin film attached. Excitation was performed using a pulsed nanoLed (455 nm), and all decay curves were obtained at room temperature.

### 2.5 Hyperspectral Fluorescence Microscopy

**Figure 1:**
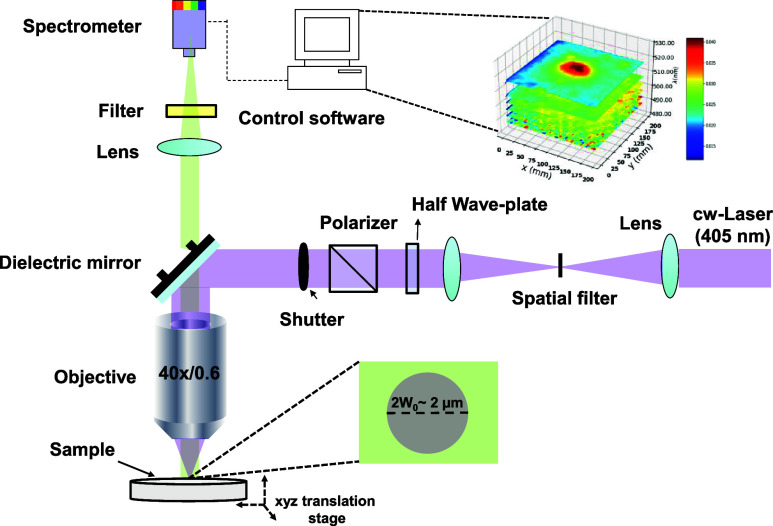
Hyperspectral fluorescence microscopy setup.

Figure 1 illustrates a schematic representation of the optical arrangement used in our hyperspectral fluorescence microscopy experiments. A continuous-wave (cw) laser emits light at 405 nm, which is directed through a spatial filter to achieve the TEM_00_ mode. Subsequently, a telescope collimates and expands the laser beam, covering the entire entrance of the microscope objective and creating a focal beam waist near the diffraction limit, thereby maximizing the peak intensity. The 405 nm beam passes through a half-wave plate and a calcite polarizer to control incident power without altering the polarization state or beam position.

The laser beam is then directed downward by a 405 nm dielectric mirror toward the microscope objective. This objective lens possesses a 40× magnification, a numerical aperture of 0.65, and a working distance of 0.6 mm. As the light beam is focused on the sample (the beam waist radius *w*_0_ = 2 μm was obtained from the zero-damage method)^23^ put on the *XYZ* translation stage (with a resolution of <1 μm), it is absorbed, leading to fluorescence emission. The objective lens captures this fluorescence, initiating the reverse optical path. The collected fluorescence is directed to the dielectric mirror, which allows fluorescence transmission while reflecting the laser excitation. Subsequently, the fluorescence passes through a 405 nm filter and converging lens, focusing the beam onto an optical fiber connected to a portable spectrometer. The entire optical system is controlled by dedicated software. All measurements were performed at room temperature (293 K) and in an air-saturable atmosphere.

## 3 Theoretical Section

To ascertain the laser-induced temperature in the spin-coated nanocrystalline CsPbBr_3_ perovskite film, we utilized the finite-difference method.^24,25^ During continuous-wave operation, the efficient conversion of the laser beam energy absorbed by material lattice electrons directly into heat allows the determination of the local temperature (*T*) at the specific time (*t*) across the two spatial dimensions (*x*, *y*) of the irradiated thin film, as described by the classical Fourier heat equation:^26^
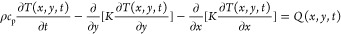
1

in which ρ = 4730 kg/m^3^^27,28^ is the average density of nanocrystals, *c*_p_ = 300 J/(kg·K)^29^ is the specific thermal capacity, and *K* = 0.43 W/(m·K)^29,30^ is the thermal conductivity of polycrystalline bulk CsPbBr_3_. Furthermore, the volumetric heat source generated by the laser beam incident on the top surface of the material is given by
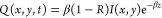
2

in which *R* is the reflectance, β (405 nm) is the absorption coefficient (4.1 × 10^6^ m^–1^),^31−33^ and *z* (∼–240 nm, obtained from the Beer Law) is the optical penetration depth at 405 nm for the thin films. As our laser beam presents a Gaussian intensity profile, we employed the following equation:
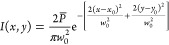
3Here, *I*(*x*, *y*) is the peak intensity (W/m^2^) at the spatial coordinate (*x*, *y*), *P̅* represents the mean laser power (W), *w*_0_ stands for the laser waist radius (m), which was estimated at ∼1 μm using the zero-damage method,^23^ and *x*_0_ and *y*_0_ indicate the grid positions where the laser is incident. At the start of the laser irradiation process, the perovskite thin film was assumed to be at room temperature (*T*(*x*, *y*, *t*_0_) = 293 K). We did not consider convection losses due to their low contribution in thin films, while the radiation losses are neglected because of the high laser intensity employed (10^6^ W/m^2^). Simultaneously, we assumed that the inherent properties of the perovskite material, including thermal conductivity, density, heat capacity, and others, remained unaltered despite temperature fluctuations. Within the finite-difference method framework, the Thomas Algorithm was used to solve the system of coupled equations and guarantee the stability condition αd*t*/(d*x*)^2^ < 1/2, in which α = *K*/ρ*c*_p_ is the thermal diffusibility (3.03 × 10^–7^ m^2^/s).^25^ The computational model was implemented in Python programming language.

## 4 Results and Discussion

**Figure 2:**
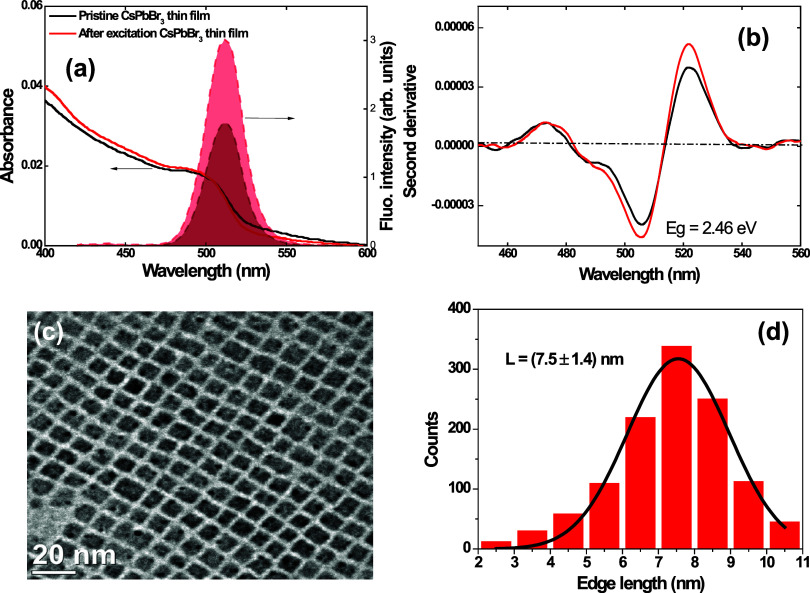
(a) Absorption (solid lines) and fluorescence (shaded curves) spectra for the CsPbBr_3_ nanocrystal thin film before and after UV lamp irradiation. (b) Second-order derivative of the absorption spectra shown in part (a). (c) TEM image and (d) particle size distribution histogram obtained from the TEM analysis.

Figure 2a displays the absorption (black solid lines) and fluorescence (black shaded curve) spectra of the pristine CsPbBr_3_ nanocrystal thin film. From these data, we calculated the bandgap from the absorption band-edge transition (black curve, Figure 2b) of the CsPbBr_3_ nanocrystal thin film from the minimum of the second-order derivative,^34,35^ i.e., *E*_g_ = [1240.7/(λ_min_)]eV = 2.46 eV. Figure 2c depicts a TEM image showcasing the cubic structure of the synthesized colloidal nanocrystals, while Figure 2d provides the corresponding particle size distribution histogram. The TEM images yielded an average edge length of *L* = (7.5 ± 1.4) nm for our cube-shaped nanocrystals, which is a size estimate consistent with the analytical empirical expression *E*_g_ = 2.25 + [1/(−1.26 + 0.996 L – 0.0324 L^2^)] reported in refs (31,32). The absorption and fluorescence spectra for the CsPbBr_3_ nanocrystals synthesized in solution and those deposited in the thin film are compared in Figure S1 of the Supporting Information (SI). Moreover, from the TEM analysis, we found that the average distance between the nanocrystals in the grid is approximately 2 nm, which is very close to the chain length of the surface capping ligands used in the synthesis procedure (oleic acid and oleylamine).^36^

**Figure 3:**
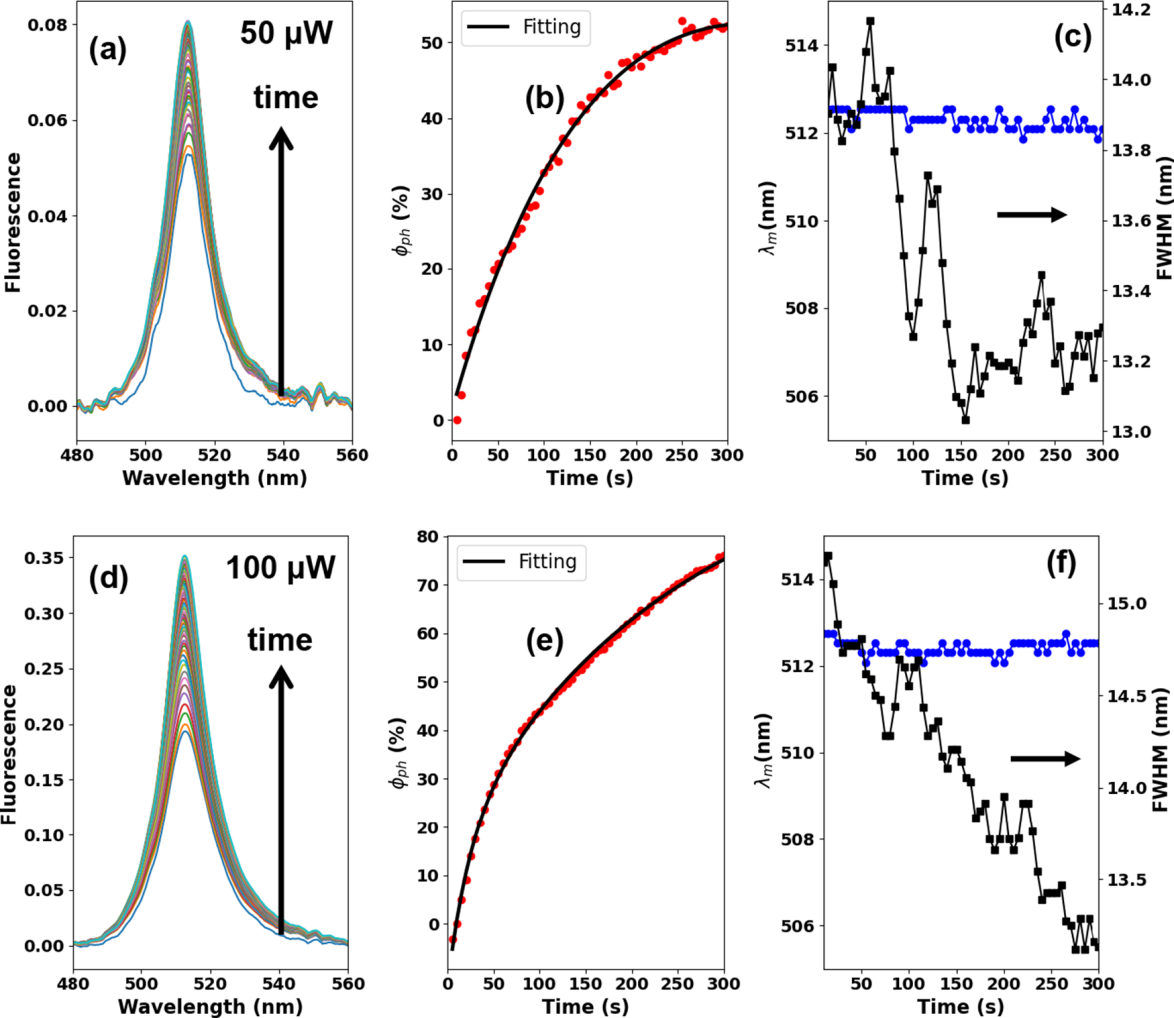
(a, d) Fluorescence spectra, (b, e) photoenhancement yield, and (c, f) emission peak wavelength (circles) and fwhm (squares) over excitation time for a CsPbBr_3_ nanocrystal thin film irradiated with a UV cw-laser at 405 nm. The result sets (a–c) and (d–f) represent the optical data obtained for the laser powers of 50 and 100 μW, respectively.

Figure 3 illustrates the fluorescence spectra (*I*_F_(λ), Figure 3a,d), photoenhancement yield (φ_ph_, Figure 3b,e), emission peak wavelength (λ_m_, Figure 3c,f, circles), and full-width at half-maximum (fwhm, Figure 3c,f, squares) over excitation time (300 s) for the prepared CsPbBr_3_ nanocrystal thin film upon irradiation with continuous-wave UV laser (405 nm) power ranging between 20 and 100 μW. We stopped the measurements at 300 s because, for higher laser powers, fluorescence photobleaching takes place after the fluorescence enhancement saturates. The irradiation experiments were performed with the following laser powers: 50 μW (Figure 3a–c) and 100 μW (Figure 3d–f).

We then monitored the temporal evolution of the emission spectrum *I*_F_(λ) under laser irradiation through a series of descriptive parameters, including the peak position and line width, and also the relative increase in the peak height , where *I*_F_^max^(*t*) is the maximum emission intensity measured at time *t* with respect to the initial instant of time *t*_0_ (before irradiation). As noted, the cw-laser irradiation increases the fluorescence intensity *I*_F_^max^ and the photoenhancement yield φ_ph_, reduces the fwhm value, while the emission peak wavelength λ_m_ remains nearly constant (∼509 nm) over the excitation time.

Comparing our two sets of results for different laser powers (50 and 100 μW), we can see that the magnitude of all these parameters tends to enhance as the laser power increases. The fwhm reduction and the fluorescence intensity rise over time, indicating that the fluorescence quantum yield of the irradiated CsPbBr_3_ nanocrystal thin film increases due to the higher laser power. We also measured the absorption spectrum after UV irradiation. Given that the fluorescence spectrum is obtained within few μm^2^ area, collecting the absorption spectrum without the contribution of film regions where the sample was not irradiated is a complicated task. To overcome this problem, the CsPbBr_3_ thin film was illuminated by a UV lamp for 10 s, as described in Section 2.3. Figure 2a compares the absorption spectrum before (black line) and after the irradiation (red line). We also measured the fluorescence spectrum at the same point, as shown in Figure 2a (red-shaded curve).

Although the UV lamp does not allow us to perform precise experimental control like the microspectroscopy setup (light intensity, temperature, focalization, etc.), it can aid us in interpreting the outcomes. First, the photoenhancement of the fluorescence intensity is clearly observed by comparing the emission spectra before (black shaded curve, Figure 2a) and after (red shaded curve, Figure 2a) the exposure to the UV lamp. Second, the bandgap value estimated from the absorption band-edge transition of the irradiated (red curve, Figure 2b) and nonirradiated (black curve, Figure 2b) film is not affected by the UV excitation process as well the average particle size. Furthermore, the CsPbBr_3_ thin film absorption increases after UV irradiation, and the scattering for the wavelengths between 530 and 600 nm is reduced.

**Figure 4:**
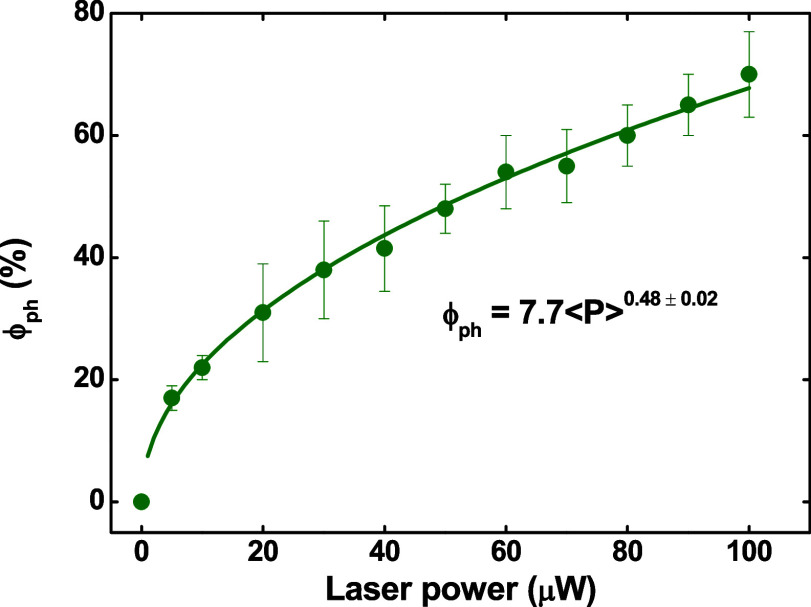
Photoenhancement yield as a function of the laser power (405 nm excitation wavelength) observed in the CsPbBr_3_ nanocrystal thin film.

Herein, we have focused on further understanding these effects based on the interplay between the photo and thermal activation. First of all, we have calculated the fluorescence photoenhancement yield (φ_ph_) at an excitation time of 300 s and observed that this quantity depends on the laser power according to the simple power law φ_ph_ = 7.7⟨P⟩^0.47±0.02^. The data are depicted in Figure 4. For laser powers higher than 150 μW, we have observed that fluorescence photobleaching takes place on the sample (fluorescence intensity reduction), as observed in other studies.^37−39^

To shed more light on these outcomes, we calculated the activation energy (*E*_a_) from the Arrhenius equation, *k* = *A*e^–E_a_/k_B_T^, in which *k* is the photoenhancement rate constant, *A* is the pre-exponential parameter, *k*_B_ is the Boltzmann constant, and *T* is the laser-induced temperature necessary to enhance the fluorescence of the CsPbBr_3_ nanocrystal thin film. The photoenhancement rate was obtained from our results by fitting the φ_ph_ data in Figure 3b,e.

It is worth mentioning that the photoenhancement rate has a monoexponential behavior (Figure 3b) for low laser power (<50 μW) and a bi-exponential behavior (φ_ph_ = *A*_1_exp(±*k*_1_*t*) + *A*_2_exp(±*k*_2_*t*)) for higher laser power (Figure 3e), which is characterized by fast and slow rate constants, represented by *k*_1_ and *k*_2_, respectively. Most probably, the bi-exponential behavior is associated with two different mechanisms. However, it is difficult to discriminate the fast and slow components in the sample because any irregularity in the thin film completely changes the rates. In this case, we choose to compute the average rate constant given by <*k*> = (A_1_*k*_1_ + A_2_*k*_2_)/(*A*_1_ + *A*_2_). On the other hand, the laser-induced final temperature was found from a simulation of the heat propagation in the irradiated thin film using the Fourier Law and the finite-difference method. The computational details can be found in Section 3. In order to validate our computational model, we compared the laser-induced temperature obtained from our simulations with experimental results reported in ref.^11^ for MAPbI_3_ thin films, in which the authors also used the spin-coated method to fabricate the thin films and found a good agreement. It is worth mentioning that the thermal diffusibility values for CsPbBr_3_ and MAPbI_3_ are similar.^29^ These data can be found in Figure S2 of the SI.

**Figure 5:**
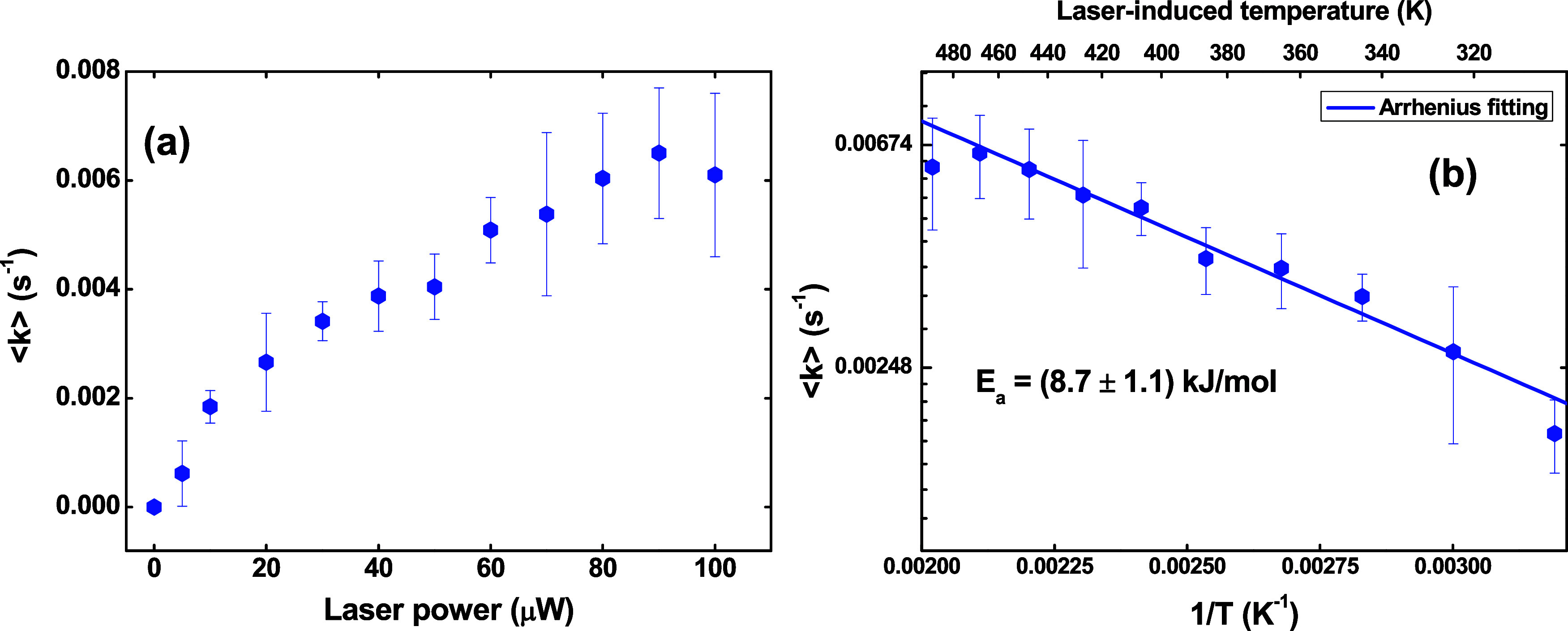
(a) Photoenhancement rate as a function of the laser power at 405 nm. (b) Photoenhancement rate over the reciprocal temperature (log–linear scale, Arrhenius plot).

Figure 5a depicts the average photoenhancement rate constant <*k*> for the CsPbBr_3_ nanocrystal thin film as a function of the laser power. From these data, we presented in Figure 5b the <*k*> values over the laser-induced reciprocal temperature 1/*T* (called the Arrhenius plot) and obtained the following estimate for the activation energy: *E*_a_ = (8.7 ± 1.1) kJ/mol.

This value is very small. Comparatively, such a value is 3–5 times lower than the activation energy for the mixed halide perovskite phase segregation (28.9 kJ/mol),^16^ a very common effect induced by light in perovskite. Therefore, the threshold laser power to observe the photoenhancement of the fluorescence intensity in our nanocrystalline perovskite film is very low. This effect occurs with a certain average rate <*k*> even at low laser-induced temperatures. Therefore, the fluorescence enhancement observed in the CsPbBr_3_ nanocrystal thin film is not exclusively related to the laser-induced annealing. According to ref (40), the increase in temperature caused by external heat sources promotes a drastic reduction in the photoluminescence intensity of CsPbBr_3_ nanocrystal thin films. They observed that at high temperatures (>400 K), the bandgap and fluorescence line width broadening become higher, reducing the radiative emission rate. In this case, the fluorescence line width broadening occurs due to the acoustic phonon–exciton coupling and longitudinal optical phonon–exciton coupling.^40^

As mentioned before, the fluorescence photoenhancement observed here should be associated with an interplay between the photo and thermal activation. In this context, we have employed hyperspectral fluorescence microscopy to map the fluorescence photoenhancement and determine its microscale length. The fluorescence mapping was performed after the laser irradiation for 300 s around the irradiated region (200 × 200 μm).

**Figure 6:**
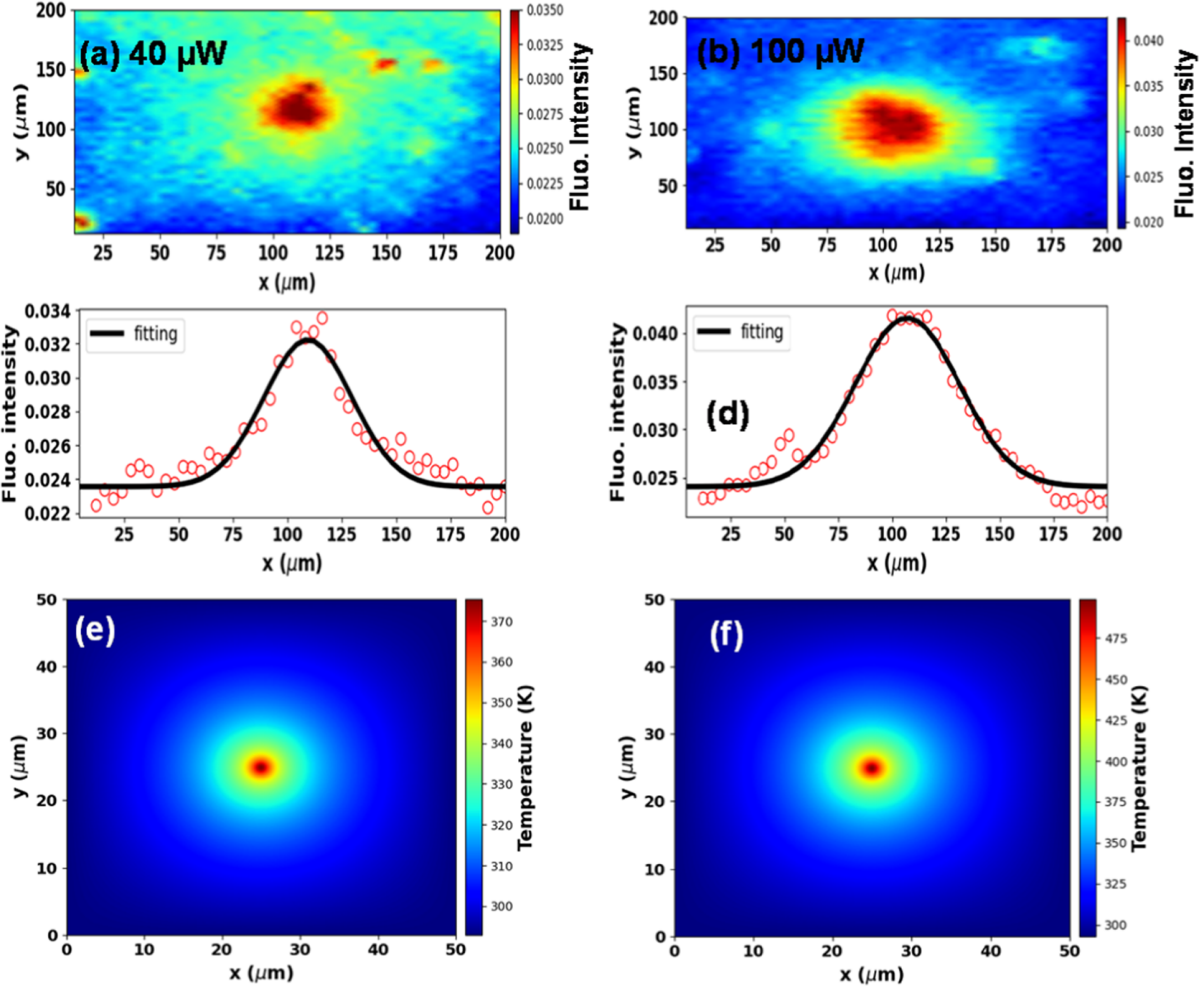
Colormaps representing the fluorescence peak intensity covering an area of 200 × 200 μm for an average laser power of (a) 40 μW and (b) 100 μW. (c, d) Corresponding fluorescence intensity versus *x*-axis translation curves at *y* = 100 μm. The solid lines represent the Gaussian fits obtained from eq 3. (e–f) Computational simulation for the heat propagation in the CsPbBr_3_ thin films for the 40 and 100 μW laser power.

Figure 6a,b shows the colormaps representing the fluorescence peak intensity covering an area of 200 × 200 μm for the laser powers of 40 and 100 μW, respectively. The (c) and (d) parts illustrate the corresponding fluorescence intensity profiles as an *x*-axis translation length function (y coordinate fixed at 100 μm). To remove any problem with the fluorescence scattering, the hyperspectral images were performed with low laser power and fast scanning laser (200 ms acquisition for each spectrum) to avoid any modification on the sample over time. As can be seen, each colormap exhibits a fluorescence photoenhancement halo similar to the thermal effects (Gaussian-like behavior). In this context, we take into account Fick’s second law of diffusion, which presents the same solution as the Fourier heat equation, to calculate the photothermal diffusion length (*L*_d_) by using the following relationship^41^:
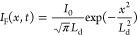
4

in which *I*_F_(*x*, *t*) is the fluorescence signal along space and time,  (α is the diffusion coefficient or thermal diffusibility (m^2^/s), *t* = τ is the fluorescence lifetime for the electronic effect or *t* = 1/⟨*k*⟩ for the photothermal effect), and *I*_0_ is a fitting parameter related to the fluorescence signal at *x* = 0. In this context, we have measured the fluorescence lifetime before and after the excitation using a UV lamp for 10 s, as described in the Experimental Section. According to ref (22), the fluorescence lifetime of the CsPbBr_3_ nanocrystals has two channels related to the hole (or electron)-trap-assisted recombination and excitonic recombination. As shown in Figure S3, the trap time is around 2.4 ns, while the exciton recombination time increases from 8.1 to 8.7 ns, indicating that the UV excitation reduces the defect levels and increases the radiative rate. Moreover, the average fluorescence lifetime increased from 7.5 to 8.1 ns after the UV lamp excitation.

**Figure 7:**
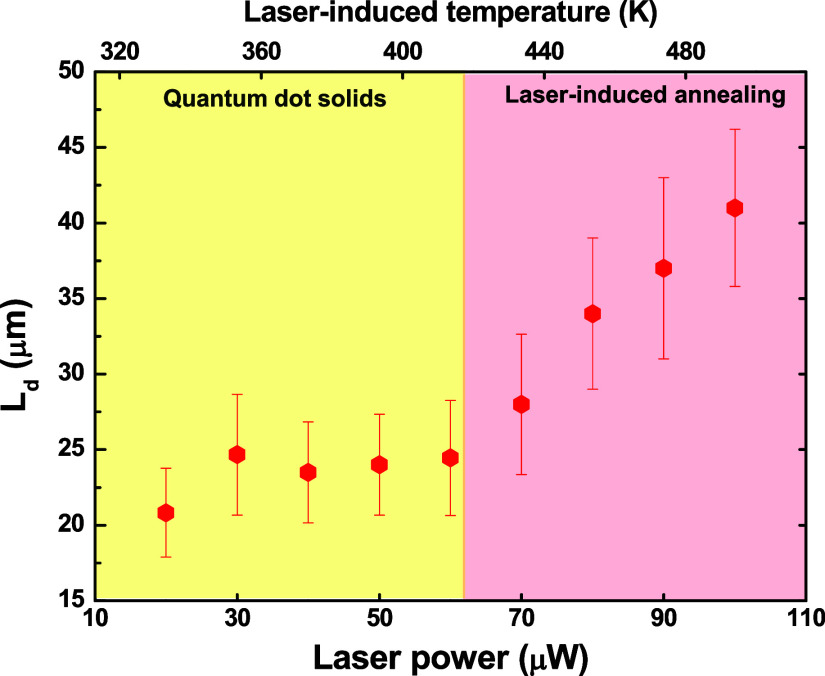
Photothermal diffusion length as a function of the laser power.

Figure 7 illustrates the photothermal diffusion length as a function of the laser power. First, the diffusion length for all laser powers is on the order of tens of micrometers, confirming that the photoenhancement phenomenon has a strong thermal character because electronic effects or even polaron generation manifest on a much smaller scale, typically submicrons to a few microns.^13,42−44^ For instance, we calculated the expected value () of the halide diffusion length due to the purely electronic event and found *L*_Br_ ≃ 100 nm. In Figure 6e,f, we illustrated the computational simulation outcomes using the finite difference method (see Section 2) for the heat propagation in the CsPbBr_3_ thin film. As observed, the fluorescence and heat profiles are similar in shape. However, the thermal profile of the excitation halo is very similar at distinct laser powers, in contrast to what we observed for the fluorescence profile. These results suggested that the structural architecture of the thin films changed, which the computational simulation does not take into consideration.

Regarding the structural change, we observe two distinct trends in the behavior of the diffusion length concerning laser power. Initially, within the range of 20–60 μW, the *L*_d_ value remains nearly constant. However, when the laser power surpasses 60 μW, a significant increase in *L*_d_ becomes evident. This transition is illustrated in Figure 7.

**Figure 8:**
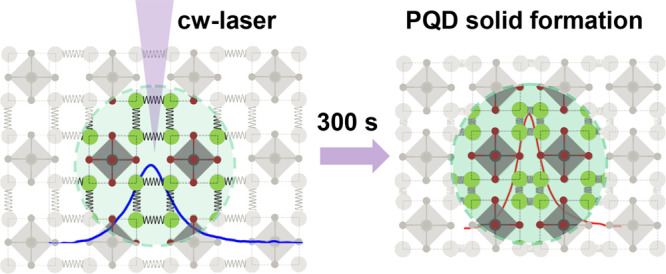
Representative diagram for laser-induced perovskite quantum dot (PQD) solid formation. After laser excitation for 300 s, the interdot distances reduce, promoting higher wave function overlap and increasing the fluorescence intensity.

At the same time, as shown in Figure 3, photoenhancement kinetic curves for laser powers below 60 μW present a monoexponential behavior, while higher laser power levels exhibit a biexponential pattern, described by characteristic fast and slow components. Consequently, the rapid component can be attributed to the reduction of the surface ligand length or, potentially, ligand detachment due to the laser-induced temperature. It is worth mentioning that the surface ligands do not absorb photons from the excitation laser (405 nm). In this case, the perovskite nanocrystals absorb light and generate heat waves that interact with the surface ligands, changing their molecular structure. This, in turn, facilitates the structural reorganization of nanocrystals, allowing them to attain a configuration resembling quantum dot solids (see Figure 8).^45−47^

Quantum dot solids exhibit ordered structures determined by the composition, size, and shape of the constituent quantum dots (QDs) within the solid matrix. These structures are typically created through precise control of the interdot distances via ligand engineering techniques.^48^ In this specific context, a reduction in interdot spacing leads to enhanced interactions between the electronic wave functions of the QDs, resulting in a cooperative effect with remarkable optical properties. Notably, this modification results in a higher absorption coefficient within the quantum dot solids (as shown in Figure 2a), thus elucidating the substantial increase in the fluorescence amplitude observed in our irradiated CsPbBr_3_ nanocrystal thin film (see Figure 8).

Conversely, the slower component of the analyzed photoenhancement behavior can be attributed to several processes, including the coalescence of nanocrystals, diffusion, and recrystallization, all of which are driven by laser-induced annealing.^11,21^ In fact, above 60 μW, the temperature in the irradiated thin film achieved values higher than 400 K, which corresponds to the annealing temperature for perovskite crystals, according to ref (11). This transformative process fosters the creation of nanocrystal superclusters, consequently leading to an observable augmentation in the photothermal diffusion length. Such an effect has already been demonstrated for the perovskite single crystals, as reported in ref (11).

**Figure 9:**
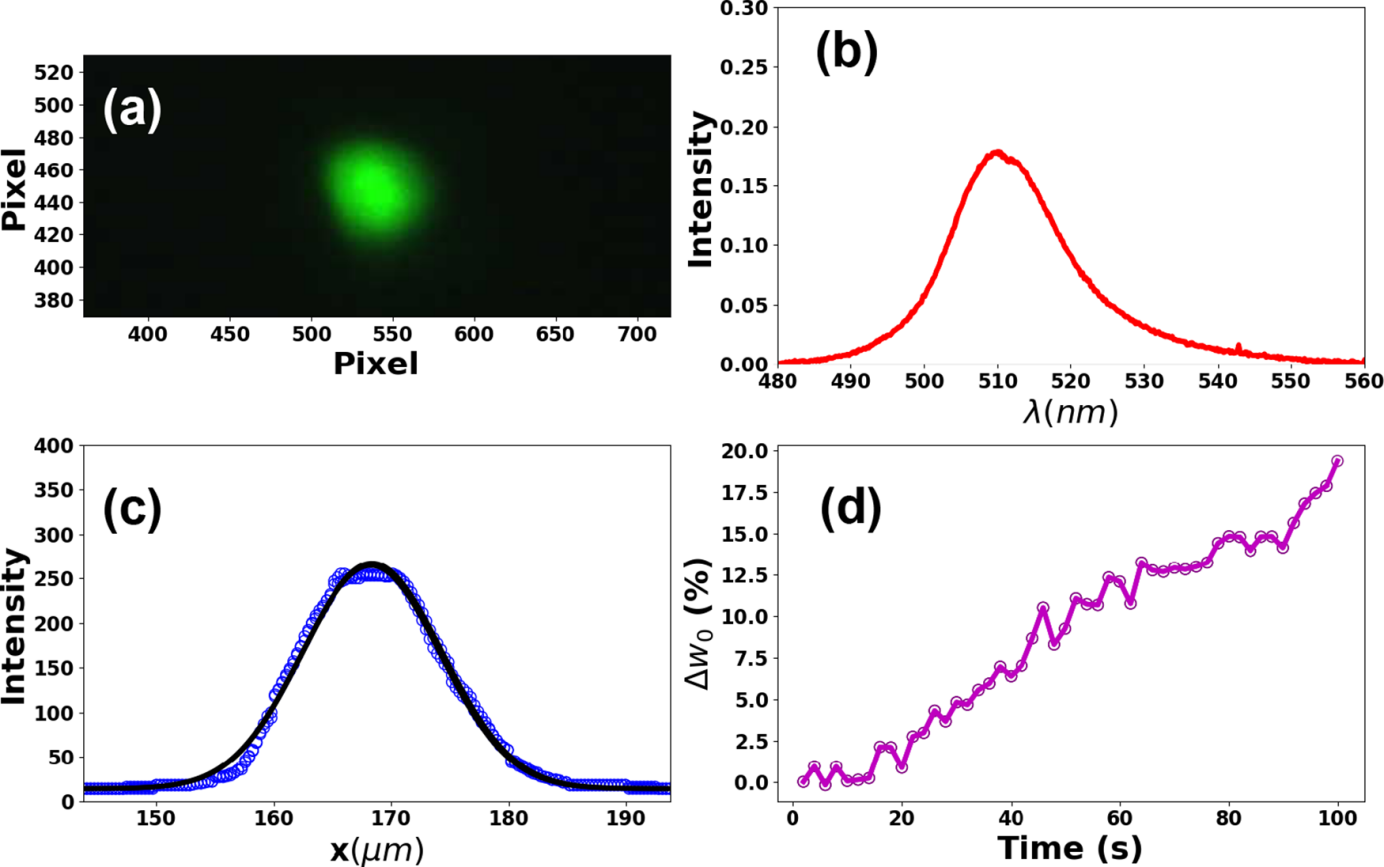
(a) Fluorescence spot after 100 s of excitation. (b) Fluorescence and (c) spatial (dots) profiles corresponding to the fluorescence spot shown in (a). (d) Change in the fluorescence spot size over time. Each spot size value was obtained by fitting the fluorescence spatial profile measured at a given excitation time, as performed in (c) for the specific time of 100 s (the solid line represents the fitting curve).

To provide further insight into our findings, we present real-time observations of fluorescence spot progression in the Supplementary video. It is important to emphasize that the camera was positioned slightly outside the objective focus in order to verify the laser effect on the change in the fluorescence spot size over time. The conclusive data from this video are represented in Figure 9, with Figure 9a showcasing the ultimate fluorescence spot (laser excitation time of 100 s). Additionally, Figure 9b,c presents fluorescence and spatial profiles (dots), respectively, corresponding to the fluorescence spot depicted in Figure 9a. The fluorescence spot size change (Δ*w*_0_) relative to the initial excitation time (*t* = 2 s), derived from fluorescence data fitting shown in Figure 9c (solid line) over excitation time, is illustrated in Figure 9d. As seen, Δ*w*_0_ exhibits an increase over the excitation time. These compelling results align closely with the data observed in hyperspectral images (Figure 6).

Now, let us look more carefully at the results reported in Figure 4. We have identified a noteworthy relationship, φ_ph_ = 7.7⟨*P*⟩^0.47±0.02^, which warrants closer examination. Imagine the quantum dot solids architecture as a superlattice (as represented in Figure 8), with the ligand acting as a spring that links the nanocrystals together or interacts with one another. We can propose a straightforward model based on harmonic oscillation to explain the extent of ligand displacement induced by temperature changes. In this scenario, the average energy for the harmonic oscillator (HO) can be expressed as <Δ*E*> = 1/2 *k*Δ*x*^2,49^ where k represents the ligand strength and Δx denotes the ligand compression caused by temperature variations. Now, let us redefine the φ_ph_ power law as  incorporating the HO energy. Thus, we arrive at the equation φ_ph_ = −(7.7k^1/2^/(2⟨Δ*t*⟩)^1/2^)(*L*–*L*_eq_), where *L*_eq_ describes the equilibrium average interdot distance (approximately 2 nm, corresponding to the length of the oleic acid/oleylamine ligand chain), and *L* represents the average distance between dots after laser excitation during the time interval Δ*t*. Given that the φ_ph_ parameter in Figure 4 is normalized by the fluorescence intensity before the irradiation, that is, without ligand compression, our HO-based model yields . Then, we can deduce *L* = 0.6 nm using the photoenhancement efficiency at the 100 μW laser power (∼70%, Figure 4), which is a plausible result given the simplicity of the model. Indeed, the estimated *L* value indicates that the surface capping ligands are subjected to a high degree of compression due to laser irradiation, as verified by the resulting fractional change in the average interparticle distance Δ*L*/*L*_eq_ = −0.7. As a consequence of the strongly reduced interparticle spacing, the nanocrystals are closely packed, thus reinforcing our previous analysis, in which the formation of a quantum dot solid-like arrangement was hypothesized.

## 5 Conclusions

In this study, we have provided insights into the mechanisms behind the fluorescence enhancement observed in CsPbBr_3_ nanocrystal thin films upon UV cw-laser illumination. Our findings suggest that this enhancement stems from the interplay between photoactivation and thermal processes. We have quantified the activation energy required for the fluorescence enhancement to occur, yielding a value of (8.7 ± 1.1) kJ/mol, notably smaller than the energy barrier observed in perovskite bulk crystals during halide phase segregation.

Additionally, our investigation involved quantifying the kinetics of fluorescence enhancement and microscale length changes. For this purpose, we combined experimental and theoretical methods such as hyperspectral fluorescence microspectroscopy and a computational thermodynamic model to correlate the photoenhancement rates at distinct laser powers with the temperature locally induced by the incident laser and the photothermal diffusion length. Our results indicate that the increase in temperature induced by the laser leads to a reduction in the interparticle spacing, achieved through the detachment of surface ligands from the nanocrystals. This reduction fosters a more pronounced overlap between the electronic wave functions of the nanocrystals, facilitating the formation of an ordered structure consistent with the so-called quantum dot solid architecture. At higher laser power levels, the coalescence, diffusion, and recrystallization of nanocrystals give rise to the formation of superclusters, as evidenced by the increased photothermal diffusion length. All these effects collectively contribute to higher radiative decay rates and, consequently, an elevated fluorescence quantum yield in these new superlattice materials.

## Supporting Information Available

Real-time fluorescence spot progression and intensity enhancement observations (MP4)
Absorption and PL spectra of the CsPbBr_3_ colloidal nanocrystal solution and the spin-coated nanocrystalline thin film; comparison of the laser-induced temperature obtained through our computational model and experimental results; and fluorescence decay curves before and after irradiation (PDF)
