# Supplementary material for: Probing the cw-Laser-Induced Fluorescence Enhancement in CsPbBr3 Nanocrystal Thin Films: An Interplay between Photo and Thermal Activation
Source: ACS Appl Mater Interfaces. 2024 Jun 17;16(26):34303–12. doi: 10.1021/acsami.4c03934 (PMC11231974; doi:10.1021/acsami.4c03934)
Supplement: Supplementary file 2 — am4c03934_si_002.pdf [file am4c03934_si_002.pdf]

Supporting information

Probing the cw-Laser Induced Fluorescence Enhancement in $CsPbBr_3$ Nanocrystal Thin Films: An Interplay Between Photo and Thermal Activation

Gabriel Fabrício de Souza,[1] Letícia Ferreira Magalhães,[2] Thaís Adriany de Souza Carvalho,[2] Diego Lourençoni Ferreira,[1] Richard Silveira Pereira, [1] Thiago Rodrigues da Cunha, [1] Jefferson Bettini, [3] Marco Antônio Schiavon,[2] Marcelo Gonçalves Vivas[1]*

[1] Laboratório de Espectroscopia Óptica e Fotônica, Universidade Federal de Alfenas, 37715-400 Poços de Caldas, MG, Brazil

[2]Grupo de Pesquisa em Química de Materiais, Universidade Federal de São João del-Rei, 36301-160 São João del-Rei, MG, Brazil

[3]Laboratório Nacional de Nanotecnologia, Centro Nacional de Pesquisa em Energia e Materiais, 13083-970 Campinas, São Paulo, Brazil

*Corresponding author: *mavivas82@gmail.com*

I.1 – Absorption and PL

Figure S1 compares the absorption and PL spectra of CsPbBr3 dispersed in colloidal solution and deposited in thin films. As can be seen, there are slight differences between the absorption and PL peaks. We observed a slight red shift in the PL from 508 to 512 nm for the thin film (red-shaded curve) concerning the colloidal solution (blue-shaded curve). Otherwise, the first excitonic transition peak occurs at the same wavelength for the absorption spectrum, but there is an absorption lineshape broadening characteristic of the little agglomerate formation.

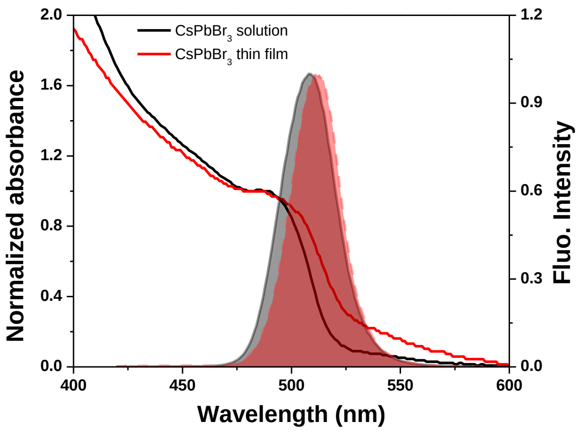

Figure S1 – Absorption and PL spectra of the $CsPbBr_3$ colloidal nanocrystal solution (Abs: black curve; PL: black shaded curve) and the spin-coated nanocrystalline thin film (Abs: red curve; PL: red shaded curve).

I.2 – Laser-induced temperature

Figure S2 compares the laser-induced temperature obtained through our computational model and experimental results reported in Ref. [1] for $MAPbI_3$ thin films. It is worth mentioning that the thermal diffusibility values for $CsPbBr_3$ and $MAPbI_3$ are similar and the authors also used the spin-coated method to fabricate the thin films.[2] Experimental data employed for the laser intensity: P = 150 mW, while $w_0 = 0.25 \pm 0.10$ mm was estimated from Fig. SI.4 (given in Ref. [1]). $I = 2.5(2P/\pi w_o^2)$, in which the prefactor 2.5 leads into consideration the translation speed of 25 mm/min.

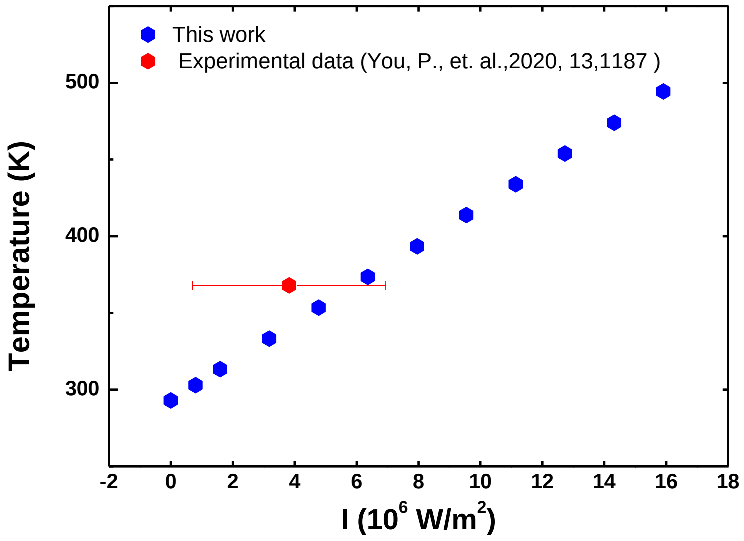

Figure S2 – Comparison of the laser-induced temperature obtained through our computational model and experimental results reported in Ref. [1] for $MAPbI_3$ thin films.

I.3 – Fluorescence lifetime

We have measured the fluorescence lifetime before and after the excitation using a UV lamp for 10 seconds, as described in the Experimental Section. According to Ref. [3], the fluorescence lifetime of the $CsPbBr_3$ nanocrystals has two channels related to the hole (or electron)-trap-assisted recombination and excitonic recombination. As shown in Fig. SI.3, the trap time is around 2.4 ns, while the exciton recombination time increases from 8.1 ns to 8.7 ns, indicating that the UV excitation reduces the defect levels and increases the radiative rate. Moreover, the average fluorescence lifetime increased from 7.5 ns to 8.1 ns after the UV lamp excitation.

0 s				
A_1 / %	τ_1 / ns	A_2 / %	τ_2 / ns	$\tau_{médio}$ / ns
28,4	2,3	71,5	8,1	7,5

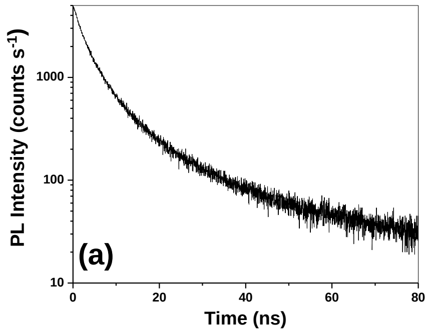

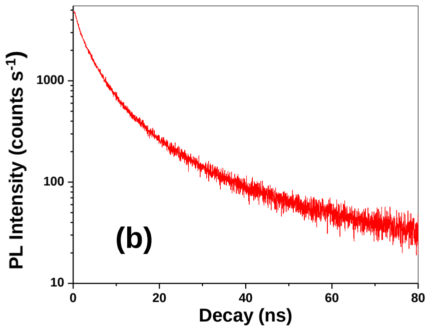

10 s				
A_1 / %	τ_1 / ns	A_2 / %	τ_2 / ns	$\tau_{médio}$ / ns
27,4	2,4	72,6	8,7	8,1

Figure S3 – Comparison of the fluorescence lifetime before (a. t=0s) and after UV-lamp irradiation (b, t=10 s).

References

(1) You, P.; Li, G. J.; Tang, G. Q.; Cao, J. P.; Yan, F. Ultrafast laser-annealing of perovskite films for efficient perovskite solar cells. *Energy & Environmental Science* **2020**, *13* (4), 1187-1196. DOI: 10.1039/c9ee02324k.

(2) Haeger, T.; Heiderhoff, R.; Riedl, T. Thermal properties of metal-halide perovskites. *Journal of Materials Chemistry C* **2020**, *8* (41), 14289-14311. DOI: 10.1039/d0tc03754k.

(3) Bonato, L. G.; Dal Poggetto, G.; Moral, R. F.; Vale, B. R.; Germino, J. C.; Almeida, D. B.; Santiago, P.; Fernandez, P. S.; Tormena, C. F.; Padilha, L. A.; et al. Photostability of amine-free CsPbBr3 perovskite nanocrystals under continuous UV illumination. *Journal of Materials Chemistry C* **2023**, *11* (24), 8231-8242. DOI: 10.1039/d2tc04953h.
